# Supplementary material for: Bioaccumulation Levels and Potential Health Risks of Mercury, Cadmium, and Lead in Albacore (Thunnus alalunga, Bonnaterre, 1788) from The Aegean Sea, Greece
Source: Int J Environ Res Public Health. 2019 Mar 6;16(5):821. doi: 10.3390/ijerph16050821 (PMC6427763; doi:10.3390/ijerph16050821)
Supplement: Supplementary file 1 [file ijerph-16-00821-s001.pdf]

Supplementary Materials

Bioaccumulation Levels and Potential Health Risks of Mercury, Cadmium, and Lead in Albacore (*Thunnus alalunga*, Bonnaterre, 1788) from The Aegean Sea, Greece

Nikolaos Stamatis *, Nikolaos Kamidis, Pelagia Pigada, Despoina Stergiou and Argyris Kallianiotis

Hellenic Agricultural Organisation—Demeter, Fisheries Research Institute, N. Peramos, Kavala 64007, Greece; nikkami@inale.gr (N.K.); pigada@inale.gr (P.P.); stergiou@inale.gr (D.S.); homekall@otenet.gr (A.K.)

* Correspondence: nikstam@inale.gr; Tel.: +30-25940-22694

Table S1. Biological data of NASSA and SASSA *T. alalunga* samples.

Species	NASSA samples					SASSA samples				
	Sample code	Fish length (cm)	Fish weight (kg)	Sex	Maturity stage	Sample code	Fish length (cm)	Fish weight (kg)	Sex	Maturity stage
T. alalunga	NASSA-1	82.4	8.528	M	2	SASSA-1	71.0	6.600	F	4
T. alalunga	NASSA-2	86.2	10.706	M	2	SASSA-2	80.0	8.800	M	5
T. alalunga	NASSA-3	90.0	10.797	M	2	SASSA-3	68.0	4.920	F	5
T. alalunga	NASSA-4	87.2	10.900	F	3	SASSA-4	71.0	6.040	F	5
T. alalunga	NASSA-5	88.7	10.368	M	2	SASSA-5	76.0	6.950	M	5
T. alalunga	NASSA-6	82.0	9.267	F	3	SASSA-6	68.0	5.030	F	5
T. alalunga	NASSA-7	87.9	10.636	M	2	SASSA-7	66.4	5.160	M	4
T. alalunga	NASSA-8	89.8	10.847	M	2	SASSA-8	77.0	6.800	M	4
T. alalunga	NASSA-9	86.0	9.164	M	2	SASSA-9	80.0	9.200	F	5
T. alalunga	NASSA-10	88.2	10.294	M	2	SASSA-10	80.0	8.600	M	4
T. alalunga	NASSA-11	85.5	9.490	F	2	SASSA-11	57.0	3.070	F	5
T. alalunga	NASSA-12	86.2	10.129	F	2	SASSA-12	70.0	5.500	M	5
T. alalunga	NASSA-13	79.0	8.448	M	2	SASSA-13	67.0	5.250	F	5
T. alalunga	NASSA-14	84.8	8.698	M	2	SASSA-14	73.0	6.380	M	4

Table S1. *Cont.*

T. alalunga	NASSA-15	81.9	8.712	M	2	SASSA-15	76.0	7.500	M	5
T. alalunga	NASSA-16	84.0	9.748	F	2	SASSA-16	73.0	6.600	M	5
T. alalunga	NASSA-17	83.1	8.540	F	2	SASSA-17	72.0	6.640	M	5
T. alalunga	NASSA-18	82.9	9.374	F	2	SASSA-18	76.0	7.180	M	5
T. alalunga	NASSA-19	87.3	10.224	M	2	SASSA-19	69.0	5.040	F	5
T. alalunga	NASSA-20	77.8	7.458	F	2	SASSA-20	70.0	6.300	M	5
T. alalunga	NASSA-21	87.2	10.617	M	2	SASSA-21	74.0	7.350	M	5
T. alalunga	NASSA-22	87.0	9.549	M	2	SASSA-22	72.0	6.250	M	5
T. alalunga	NASSA-23	83.2	9.900	M	2	SASSA-23	60.0	3.800	M	5
T. alalunga	NASSA-24	86.4	9.016	M	2	SASSA-24	66.0	4.390	F	5
T. alalunga	NASSA-25	90.3	11.438	F	3	SASSA-25	74.0	6.380	M	5
T. alalunga	NASSA-26	98.2	11.096	M	2	SASSA-26	64.0	4.800	M	5
T. alalunga	NASSA-27	82.2	8.678	F	3	SASSA-27	66.0	3.990	F	5
T. alalunga	NASSA-28	95.5	12.518	M	2	SASSA-28	68.0	5.230	M	5
T. alalunga	NASSA-29	87.9	10.885	F	3	SASSA-29	76.0	6.600	M	4
T. alalunga	NASSA-30	81.4	8.631	F	2	SASSA-30	72.0	5.400	F	5
T. alalunga	NASSA-31	92.3	12.329	M	2	SASSA-31	71.0	5.430	M	4
T. alalunga	NASSA-32	90.6	10.563	M	2	SASSA-32	65.0	4.900	F	4
T. alalunga	NASSA-33	86.3	9.876	M	2	SASSA-33	74.0	6.820	M	5
T. alalunga	NASSA-34	91.0	9.725	F	2	SASSA-34	63.0	3.660	F	5
T. alalunga	NASSA-35	89.7	8.590	F	3	SASSA-35	76.0	6.490	M	5
T. alalunga	NASSA-36	90.3	9.498	M	2	SASSA-36	73.0	6.100	F	5
T. alalunga	NASSA-37	94.7	10.569	F	3	SASSA-37	67.0	5.010	F	5
T. alalunga	NASSA-38	101.5	11.457	M	2	SASSA-38	65.0	4.400	F	4
T. alalunga	NASSA-39	99.5	11.679	M	3	SASSA-39	76.0	8.300	M	5
T. alalunga	NASSA-40	92.6	9.198	M	2	SASSA-40	66.0	4.660	F	4
T. alalunga	NASSA-41	86.8	7.568	M	2	SASSA-41	75.0	7.500	F	5

Table S2. Concentrations (mg kg^{-1} wet wt) of selected heavy metals of NASSA and SASSA *T.alalunga* samples.

Species	NASSA samples							SASSA samples						
	Sample code	Mean Hg	SD	Mean Cd	SD	Mean Pb	SD	Sample code	Mean Hg	SD	Mean Cd	SD	Mean Pb	SD
T. alalunga	NASSA-1	0.331	0.010	0.028	0.001	ND	ND	SASSA-1	0.593	0.010	0.021	0.001	0.022	0.001
T. alalunga	NASSA-2	0.360	0.019	0.029	0.001	ND	ND	SASSA-2	0.472	0.012	0.023	0.001	0.027	0.001
T. alalunga	NASSA-3	0.462	0.011	0.044	0.003	**0.557**	0.022	SASSA-3	0.426	0.015	0.061	0.003	0.029	0.001
T. alalunga	NASSA-4	0.249	0.008	0.022	0.001	0.021	0.001	SASSA-4	0.345	0.009	0.023	0.001	0.023	0.001
T. alalunga	NASSA-5	0.483	0.010	0.041	0.001	0.024	0.001	SASSA-5	0.444	0.010	0.022	0.001	0.021	0.001
T. alalunga	NASSA-6	0.321	0.010	0.066	0.003	ND	ND	SASSA-6	0.327	0.017	0.027	0.001	0.023	0.001
T. alalunga	NASSA-7	0.460	0.008	0.047	0.001	ND	ND	SASSA-7	0.369	0.013	0.029	0.001	0.061	0.006
T. alalunga	NASSA-8	0.456	0.008	0.042	0.001	0.029	0.001	SASSA-8	0.524	0.024	0.023	0.001	0.023	0.001
T. alalunga	NASSA-9	0.601	0.019	0.043	0.002	ND	ND	SASSA-9	0.724	0.027	0.023	0.001	0.047	0.002
T. alalunga	NASSA-10	0.364	0.014	0.024	0.001	0.085	0.014	SASSA-10	0.492	0.022	0.024	0.001	0.060	0.003
T. alalunga	NASSA-11	0.447	0.011	0.022	0.001	**0.488**	0.018	SASSA-11	0.141	0.008	0.025	0.001	0.049	0.002
T. alalunga	NASSA-12	0.578	0.012	0.043	0.002	0.107	0.010	SASSA-12	0.368	0.010	0.026	0.001	**0.422**	0.019
T. alalunga	NASSA-13	0.266	0.010	0.067	0.003	0.024	0.001	SASSA-13	0.242	0.007	0.027	0.001	0.069	0.007
T. alalunga	NASSA-14	0.521	0.013	0.046	0.003	0.029	0.001	SASSA-14	0.395	0.010	0.029	0.001	ND	0.000
T. alalunga	NASSA-15	0.439	0.014	0.088	0.004	ND	ND	SASSA-15	0.753	0.020	0.022	0.001	ND	0.000
T. alalunga	NASSA-16	0.365	0.015	**0.669**	0.012	0.047	0.002	SASSA-16	0.537	0.011	0.022	0.001	ND	0.000
T. alalunga	NASSA-17	0.397	0.011	**0.100**	0.001	ND	ND	SASSA-17	0.699	0.020	0.025	0.001	0.021	0.001
T. alalunga	NASSA-18	0.407	0.010	0.064	0.003	0.026	0.001	SASSA-18	0.443	0.026	0.044	0.006	0.023	0.001
T. alalunga	NASSA-19	0.448	0.018	0.049	0.001	ND	ND	SASSA-19	0.428	0.011	**0.144**	0.016	0.025	0.006
T. alalunga	NASSA-20	0.289	0.011	**0.109**	0.001	ND	ND	SASSA-20	0.633	0.017	0.089	0.004	0.046	0.015
T. alalunga	NASSA-21	0.594	0.021	**0.108**	0.001	0.045	0.002	SASSA-21	0.579	0.028	**0.185**	0.014	0.049	0.019
T. alalunga	NASSA-22	0.485	0.020	0.047	0.002	0.046	0.002	SASSA-22	0.320	0.033	0.021	0.001	ND	0.000
T. alalunga	NASSA-23	0.416	0.010	0.085	0.003	0.027	0.001	SASSA-23	0.535	0.062	0.023	0.001	0.066	0.003
T. alalunga	NASSA-24	0.595	0.022	**0.105**	0.003	ND	ND	SASSA-24	0.185	0.011	0.047	0.001	0.020	0.001
T. alalunga	NASSA-25	0.317	0.012	**0.106**	0.001	ND	ND	SASSA-25	0.418	0.020	0.063	0.005	0.046	0.006

Table S2. *Cont.*

T. alalunga	NASSA-26	0.368	0.010	**0.164**	0.001	0.028	0.002	SASSA-26	0.199	0.013	0.081	0.005	0.020	0.001
T. alalunga	NASSA-27	0.349	0.010	0.069	0.004	ND	ND	SASSA-27	0.224	0.010	0.062	0.004	0.040	0.009
T. alalunga	NASSA-28	0.720	0.027	**0.481**	0.013	ND	ND	SASSA-28	0.267	0.022	0.043	0.004	ND	0.000
T. alalunga	NASSA-29	0.569	0.022	**0.342**	0.013	0.066	0.001	SASSA-29	0.261	0.008	0.069	0.001	0.295	0.029
T. alalunga	NASSA-30	0.650	0.020	**0.543**	0.019	0.026	0.001	SASSA-30	0.278	0.010	**0.109**	0.013	0.028	0.001
T. alalunga	NASSA-31	0.571	0.020	**0.303**	0.018	ND	ND	SASSA-31	0.259	0.007	0.087	0.011	0.028	0.001
T. alalunga	NASSA-32	0.892	0.025	**0.244**	0.012	ND	ND	SASSA-32	0.271	0.010	0.046	0.001	ND	0.000
T. alalunga	NASSA-33	0.634	0.016	**0.265**	0.012	ND	ND	SASSA-33	0.238	0.020	0.063	0.003	ND	0.000
T. alalunga	NASSA-34	0.473	0.011	**0.386**	0.017	ND	ND	SASSA-34	0.251	0.011	0.021	0.001	0.049	0.003
T. alalunga	NASSA-35	0.395	0.010	**0.427**	0.016	0.029	0.001	SASSA-35	0.512	0.020	0.066	0.002	ND	0.000
T. alalunga	NASSA-36	0.516	0.016	**0.368**	0.015	0.028	0.001	SASSA-36	0.322	0.026	0.069	0.001	0.022	0.001
T. alalunga	NASSA-37	0.397	0.017	**0.347**	0.016	0.021	0.003	SASSA-37	0.485	0.009	**0.108**	0.010	ND	0.000
T. alalunga	NASSA-38	0.938	0.028	**0.322**	0.014	ND	ND	SASSA-38	0.344	0.010	**0.588**	0.042	0.108	0.020
T. alalunga	NASSA-39	0.691	0.022	**0.469**	0.013	0.221	0.012	SASSA-39	0.257	0.017	**0.141**	0.011	0.069	0.006
T. alalunga	NASSA-40	0.426	0.018	**0.427**	0.012	0.023	0.001	SASSA-40	0.334	0.010	**0.506**	0.059	0.029	0.001
T. alalunga	NASSA-41	0.456	0.016	**0.302**	0.017	0.025	0.001	SASSA-41	0.538	0.010	**0.243**	0.028	0.023	0.001

SD = Standard deviation; Bold highlighted numbers show values over the EU permissible limits.

Table S3. Spearman Rank Order Correlations between parameters of NASSA samples.

	NASSA-length	NASSA-weight	NASSA-sex	NASSA-stage	NASSA-Pb	NASSA-Cd	NASSA-Hg
**NASSA-length**	1.000						
**NASSA-weight**	**0.726**	1.000					
**NASSA-sex**	**-0.287**	-0.201	1.000				
**NASSA-stage**	0.133	0.172	**0.520**	1.000			
**NASSA-Pb**	0.096	0.106	0.054	0.030	1.000		
**NASSA-Cd**	**0.343**	0.049	0.137	0.156	-0.059	1.000	
**NASSA-Hg**	**0.383**	0.292	**-0.377**	-0.299	0.076	**0.321**	1.000

Bold highlighted numbers show positive or (-) negative significant correlation

Table S4. Spearman Rank Order Correlations between parameters of SASSA samples.

	**SASSA-length**	**SASSA-weight**	**SASSA-sex**	**SASSA-stage**	**SASSA-Pb**	**SASSA-Cd**	**SASSA-Hg**
**SASSA-length**	1.000						
**SASSA-weight**	**0.946**	1.000					
**SASSA-sex**	**0.467**	**0.492**	1.000				
**SASSA-stage**	0.012	0.026	-0.045	1.000			
**SASSA-Pb**	-0.118	-0.110	-0.077	-0.143	1.000		
**SASSA-Cd**	-0.129	0.169	-0.200	-0.094	0.185	1.000	
**SASSA-Hg**	**0.486**	**0.563**	0.241	0.010	-0.106	-0.231	1.000

Bold highlighted numbers show positive or (-) negative significant correlation.
